# The future is coming: promising perspectives regarding the use of machine learning in renal transplantation

**DOI:** 10.1590/2175-8239-JBN-2018-0047

**Published:** 2018-10-18

**Authors:** Pedro Guilherme Coelho Hannun, Luis Gustavo Modelli de Andrade

**Affiliations:** 1 Universidade Estadual Paulista Departamento de Medicina Interna São PauloSP Brasil Universidade Estadual Paulista, Departamento de Medicina Interna, São Paulo, SP, Brasil.

**Keywords:** Machine Learning, Kidney Transplantation, Models, Statistical

## Abstract

**Introduction:**

The prediction of post transplantation outcomes is clinically important and involves several problems. The current prediction models based on standard statistics are very complex, difficult to validate and do not provide accurate prediction. Machine learning, a statistical technique that allows the computer to make future predictions using previous experiences, is beginning to be used in order to solve these issues. In the field of kidney transplantation, computational forecasting use has been reported in prediction of chronic allograft rejection, delayed graft function, and graft survival. This paper describes machine learning principles and steps to make a prediction and performs a brief analysis of the most recent applications of its application in literature.

**Discussion:**

There is compelling evidence that machine learning approaches based on donor and recipient data are better in providing improved prognosis of graft outcomes than traditional analysis. The immediate expectations that emerge from this new prediction modelling technique are that it will generate better clinical decisions based on dynamic and local practice data and optimize organ allocation as well as post transplantation care management. Despite the promising results, there is no substantial number of studies yet to determine feasibility of its application in a clinical setting.

**Conclusion:**

The way we deal with storage data in electronic health records will radically change in the coming years and machine learning will be part of clinical daily routine, whether to predict clinical outcomes or suggest diagnosis based on institutional experience.

## Introduction

Machine learning is a statistical technique that allows a computer to make future predictions based on experiences. Conventional statistics are structured in comparisons with samples based on a known distribution to judge whether differences in data support the existence of an effect in the population they represent. Ronald Fisher established that a probability of less than 5% is statistically significant or, in other words, that the sample studied differs from the population (*p* = 0.05)^1^. Fisher's early work was based on calculation of the standard deviation, which assumes that data are normally distributed. The normal distribution is represented as a bell-shaped curve, with the mean in the top of the bell and the "tails" falling off at the sides. Standard deviation is simply the "average" of the absolute deviation of a value from the mean and is a measurement of uncertainty. Therefore, this formal statistical approach considers that samples have a normal distribution, also known as parametric model. The next step in formal statistics is to make comparisons by using a known distribution (Student t-test, Fisher's or chi-squared distribution).

Machine learning, on the other hand, uses mainly nonlinear models, which are less dependent on the type data distribution. Some examples are decision trees, artificial neural network, k nearest neighbor's algorithm, and support vector machines^2^. The main differences between these two strategies is that conventional statistics seek to find differences between samples while machine learning is aimed at creating models that can predict future events. Machine learning techniques are focused on the importance of the predictor and the targeted response instead of turning it only to statistical analysis for *p* values.

Steps involved in machine learning can be summarized as data acquisition, cleaning and preparation, modeling, and deployment^2^. The first steps involved in data acquisition can be done automatically by using existing databases. For the second step, data management procedures such as removal of nonrelevant variables, data instantiation, missing data imputation, outlier removal, and selection of the most important variables (predictors), are usually done in preparation for the modeling stage. In the last step, several models are designed to predict the response; those that incorporate the best prediction algorithms are selected and their results are combined (ensemble model). Generally, a model is constructed with 70% of all available data, which is called "training," and the remaining 30% is test data (not used in model assembly). The test data are used in order to validate the model and avoid overfitting, defined as the production of an analysis that corresponds too closely to a particular set of data.

## Application

Because many factors influence the course of a disease, accurate prediction of whether treatment will result in one or more disease outcomes by using formal statistical patterns is difficult. New approaches, such as the use of data mining techniques, can improve precision and accuracy in predicting results of different types of treatment, with simultaneous consideration of several factors as well as complex interactions occurring between them. Machine learning, which is a major technical basis for data mining, provides a method for extracting information from raw data within medical records^3^.

Bihorac *et al*. recently reported a machine learning application in the medical field^4^. In a single center cohort of 51,457 patients, researchers developed and validated an algorithm (MySurgeryRisk) to predict probabilistic risk scores for 8 postoperative complications (acute renal injury, venous thromboembolism, intensive care admission for more than 48 hours, mechanical ventilation for 48 hours, wound, neurologic and cardiovascular complications, and death up to 24 months after surgery) by using only clinical data stored in institutional health electronic records^4^. Development of the algorithm consisted of two stages classified as data transformer and data analytics. The data transformer step integrated the available data (demographic, preoperative, socioeconomic, administrative, medical, pharmaceutical, and laboratory variables) so that the data pre-processing procedure and model selection could be performed posteriorly to optimize data analysis. The data analytics step used several computational algorithms to calculate risk probabilities for postoperative surgery and mortality for an individual patient. Finally, MySurgeryRisk was submitted to crossvalidation, that is, a model was tested in 250 different and randomly selected cohorts composed of patients within the sample (a total of 10,291 patients in each validation cohort). The results showed a predictive power for the 8 complications ranging from 0.82 to 0.94 (99% confidence interval [CIs], 0.81 to 0.94); for death, the risk at different time periods after surgery ranged from 0.77 and 0.83 (99% CI, 0.76 to 0.85)^4^.

In renal transplantation, establishing an association with clinical outcomes is even more difficult than in everyday clinical practice because of the high complexity of the treatment. An exhaustively studied issue is the prediction of long-term graft survival^5^^-^7

Over the past few decades, even though improvements in immunosuppressive therapy have significantly reduced acute rejection rates and increased short-term graft survival, substantial benefits in long-term survival did not occur. Speculated causes include influence of new donor and recipient profiles; however, so far, factors that contribute to this observation are not well defined, as is the ability to make a precise prognosis and predict duration of the transplant^6^^-^8. The current models of long-term graft survival are limited by multiple factors, including: dependence on pre-transplant factors, without consideration of immunologic factors;^6^^-^10 the relatively small sample sizes used to construct the models;^8^^-^10 and failure to accurately use censored patient data^6^^-^10. In addition, the observation time of the existing models is relatively short, whereas a long observation period would be essential to predict the long-term survival of the graft^6^^,^^7^.

In this context, Yoo *et al*. assessed the use of machine learning techniques to predict graft survival^11^. By applying data mining methods combined with survival statistics, the researchers constructed predictive models of graft survival that included immunologic factors as well as known variables of receptors and donors, following a method similar to the construction of the algorithm previously described. For that purpose, they used a retrospective data analysis from a multicenter cohort study of 3,117 patients and analyzed the predictive power of a joint learning algorithm. Subsequently, results were compared with those from conventional models. The analysis by a conventional tree model found that association of serum creatinine level 3 months after transplantation using a graft failure rate of 77.8% had a 0.7 concordance (an index that measures how well the model discriminates between different responses, that is, between the expected response and observed response). Interestingly, the use of a survival decision tree, another standard model from a data mining method, increased the concordance of the prediction in relation to the first algorithm (agreement of 0.8), including the incidence of acute rejection in the first year after transplantation^11^.

Further attempts have been made to use machine learning tools for data mining in order to predict long-term graft survival. Among these attempts, a good correlation between predicted graft survival and the observed 10-year survival rate calculated from survival data in the United States Renal Data System was identified using decision tree modeling^6^. In another modeling study that used data from 1542 kidney transplant recipients from the Dialysis and Transplantation Registry of Australia and New Zealand, the success or failure of a transplant was predicted with an accuracy of 85% using artificial neural networks^9^. Bayesian classifiers were able to predict the success or failure of the transplant with accuracy of 97%. However, prediction accuracy of long-term graft survival duration was lower in 68% of the studies^10^.

## Discussion

The consensus of most authors cited in these previous studies is similar: despite fair findings in highly representative patient groups, further research is needed to externally validate this approach, determine feasibility of its application in a clinical setting, and assess whether its use could lead to better results compared with current practices. Still, such studies consider that machine learning methods could provide flexible and workable tools to predict outcomes involving multiple variables.

Undoubtedly, evidence exists on the benefit derived from these computational techniques in the medical field, but how this evidence can be implemented in the medical routine and, more specifically, in management of renal transplantation receptors, is still unclear. In addition, what areas should these new studies address? Answers to these questions would include generation of clinical decisions based on dynamic and local practice data, as well as optimization of organ allocation and post-transplantation care.

Currently, many clinical decisions are often based on the physician's experience and intuition^5^. These common practices have led to errors and excessive medical costs that affect the quality of the service provided for patients. Machine learning methods could help generate patterns of evolution based on large local and multicenter data sets, which would be very useful to improve quality of clinical decisions. Today, a wealth of data is readily available in hospital electronic medical records and large national databases, such as those maintained by the United Network for Organ Sharing (UNOS), which is remarkably complete, and is currently not being used to benefit patients^5^. This kind of analysis could provide targeted care to particular types or subpopulations of patients at increased risk for graft loss or death. This application was shown to some extent by Taber *et al*., who found that in a risk analysis, dynamic data at the patient level improved accuracy of prediction for rehospitalization within 30 days after kidney transplantation^12^.

An even better possibility would be to develop a targeted organ allocation scheme that would focus on post-transplant outcome as a measure of performance^13^. The whole process would be based on transplant optimization to ensure that transplant would be performed only on patients who would have long-term benefit. Much of this need arises from the fact that, because of the scarcity of donor organs^5^^-^7, there is an increasing requirement for the development of effective and efficient procedures to select the ideal organ recipient and guarantee maximum possible survival. In the future, a new tool based on such techniques could be designed to assist in the complex decision-making process used to identify good transplant candidates for specific features of available kidneys.

Several predictive modeling techniques could be employed for the statistical components of the discussion such as machine support vectors, artificial neural networks, Bayes classifiers, and regression trees^6^^-^10 in order to develop predictive models and extract the most useful variables in a sensitivity analysis by using the best performance model (such as shown in previous studies). Survival analysis could be estimated using regression models based on data collected from candidates and donors (such as the Cox proportional hazards regression model); this would provide information on the survival benefit that a given transplant could provide to a patient. A critical prognostic index could be conceived, which would classify patients who undergo transplantation in terms of several risk categories (low, medium, and high), among many other possibilities^13^.

## Conclusion

A large volume of information exists in digitized format in electronic health databases. The next challenge is how to provide useful analysis of this information. Machine learning seems to be the most viable option for such analysis. The way that health professionals deal with hospital data will radically change in the coming years, and this trend is highly relevant for clinical and surgical decisions that are based on computational patterns obtained from each local practice.
